# Eculizumab Pharmacokinetics and Pharmacodynamics in Patients With Generalized Myasthenia Gravis

**DOI:** 10.3389/fneur.2021.696385

**Published:** 2021-11-02

**Authors:** Jonathan P. R. Monteleone, Xiang Gao, Huub Jan Kleijn, Francesco Bellanti, Ryan Pelto

**Affiliations:** ^1^Department of Pharmacometrics, PK/PD M&S, Clinical Development and Translational Sciences, Alexion Pharmaceuticals Inc., Boston, MA, United States; ^2^Certara Strategic Consulting, Oss, Netherlands

**Keywords:** generalized myasthenia gravis, pharmacokinetics, pharmacodynamics, complement, eculizumab, autoimmune, exposure-response analysis

## Abstract

**Objective:** To investigate the pharmacokinetics, pharmacodynamics, and exposure–response of the approved 900/1,200 mg dosing regimen for the terminal complement component 5 (C5) inhibitor eculizumab in patients with generalized myasthenia gravis (gMG).

**Methods:** The analysis used data from 62 patients aged ≥ 18 years with anti-acetylcholine receptor (AChR) antibody-positive refractory gMG who received eculizumab during the REGAIN study (ClinicalTrials.gov: NCT01997229). One- and two-compartment population-pharmacokinetic models were evaluated, and the impact of covariates on pharmacokinetic parameters was assessed. Relationships between eculizumab exposure and free C5 concentration, *in vitro* hemolytic activity, clinical response, and tolerability were characterized.

**Results:** Steady-state serum eculizumab concentrations were achieved by Week 4 and were sustained throughout the 26-week treatment period. The eculizumab pharmacokinetic data were well-described by a two-compartment model with first-order elimination, including effects of body weight on pharmacokinetic parameters and plasma-exchange events on clearance. Complete inhibition of terminal complement was achieved in nearly all patients at the time of trough and peak eculizumab concentrations at all post-dose timepoints assessed (free C5 < 0.5 μg/ml in 92% of patients; *in vitro* hemolysis < 20% in 87% of patients). Serum eculizumab concentrations of ≥116 μg/ml achieved free C5 concentrations of < 0.5 μg/ml. Clinical efficacy and tolerability were consistent across the eculizumab exposure range.

**Conclusions:** Rigorous, quantitative, model-based exposure–response analysis of serum eculizumab concentration and response data demonstrated that the approved eculizumab dosing (900/1,200 mg) for adults with anti-AChR antibody-positive refractory gMG rapidly achieved complete inhibition of terminal complement activation and provided sustained clinical efficacy across the eculizumab exposure range.

## Introduction

Eculizumab is the first complement-targeting drug approved for the treatment of complement-mediated neurological autoimmune diseases, such as myasthenia gravis (MG) and neuromyelitis optica spectrum disorder (NMOSD). It binds specifically and with high affinity to human terminal complement component C5, inhibiting its cleavage to C5a and C5b during complement activation (1). Strategic blockade of the complement cascade at C5 is associated with clinical benefits because it prevents both release of proinflammatory mediators and formation of the terminal membrane attack complex involved in autoantigen-specific destruction, while preserving the components of proximal complement activation necessary for opsonization of microorganisms and clearance of immune complexes (2). As a recombinant humanized immunoglobulin (Ig)G2/4 monoclonal antibody, eculizumab has been engineered to provide potent inhibition of the terminal complement pathway, with a low potential for eliciting immune or proinflammatory responses (2). The IgG2 and IgG4 components of eculizumab are not believed to activate the classical complement pathway, unlike IgG1 and IgG3 antibody subtypes, which are potent activators.

Complement activation is a major cause of disease pathogenesis in patients with generalized MG (gMG) (3, 4)—a chronic autoimmune disease characterized by fluctuating and often severe muscle weakness and fatigability (3, 5, 6). In the majority (~85%) of patients, it is driven by autoantibodies directed against the nicotinic acetylcholine receptor (AChR) on the postsynaptic membrane of the neuromuscular junction (7). Anti-AChR antibodies (which are primarily of subclasses IgG1 and IgG3) activate the classical complement cascade, ultimately leading to the formation of the membrane attack complex. This damages the postsynaptic membrane of the neuromuscular junction, leading to abnormal neuromuscular transmission and the characteristic symptoms of muscle weakness and fatigability. Inhibition of terminal complement activation is therefore a logical approach to prevent damage at the neuromuscular junction in patients with anti-AChR antibody-positive gMG (8).

The viability of this approach has been demonstrated by studies providing evidence that eculizumab improves clinical outcomes in adult patients with anti-AChR antibody-positive refractory gMG and is well-tolerated (9–12), which led to its approval in the USA (adults with AChR antibody-positive gMG), Canada (adults with AChR antibody-positive treatment-refractory gMG), Europe (adults with AChR antibody-positive treatment-refractory gMG), and Japan (patients with AChR antibody-positive gMG whose symptoms are difficult to control with high-dose intravenous immunoglobulin therapy or plasmapheresis) (13–16). In the 6-month, double-blind, placebo-controlled, Phase 3 REGAIN study, clinically meaningful improvements in activities of daily living, muscle strength, functional ability, and health-related quality of life were observed in patients with gMG who received eculizumab (10, 12, 17–19). These clinical improvements were sustained through 3 years of continuous treatment in the open-label extension phase of this study; patients in the open-label extension who had previously received placebo in the REGAIN study also demonstrated a rapid response to eculizumab (11, 12, 17–19).

The present analysis of data from the REGAIN study was undertaken to further explore the pharmacokinetic/pharmacodynamic profile of the approved eculizumab dosing regimen in gMG. A model-based approach was used to characterize dose–concentration and concentration–free C5 and terminal complement activity relationships for eculizumab in patients with anti-AChR antibody-positive refractory gMG, and to identify patient-related factors that might influence these relationships. The relationships between eculizumab exposure and (i) clinical effect, (ii) adverse events (AEs), and (iii) the potential development of anti-drug antibodies (ADAs) were investigated.

## Methods

### Study Population and Study Design

This analysis was based on pharmacokinetic, pharmacodynamic, efficacy, and safety data from the randomized, double-blind, placebo-controlled, multicenter, Phase 3 REGAIN study (ClinicalTrials.gov identifier: NCT01997229) of eculizumab in patients with anti-AChR antibody-positive refractory gMG. Refractory gMG was defined as two or more immunosuppressive therapies (including corticosteroids), or at least one immunosuppressive therapy with intravenous immunoglobulin or plasma exchange given at least four times per year, for 12 months without symptom control.

The REGAIN study population comprised 125 patients aged ≥ 18 years with anti-AChR antibody-positive refractory gMG, of whom 62 patients received intravenous eculizumab for 26 weeks, initiated at 900 mg weekly (every 7 ± 2 days) for the first four doses (starting on Day 1), followed by 1,200 mg 1 week later at Week 4 (the fifth dose) and then every 2 weeks (14 ± 2 days) thereafter. The remaining 63 patients received placebo.

### Sample Collection

Each intravenous eculizumab dose was administered over ~35 min. Blood samples for measurement of baseline and trough eculizumab concentrations, *in vitro* hemolytic activity (as a measure of terminal complement activity), and serum free C5 concentrations were taken 5–90 min before infusion of eculizumab at baseline and at Weeks 1, 4, 8, 12, 20, and 26. ADA titers were measured in the blood samples taken at baseline and at Weeks 4, 12, and 26. Blood samples for measurement of peak eculizumab concentrations, *in vitro* hemolytic activity, and serum free C5 concentrations were taken at least 60 min after the eculizumab infusion was completed at the first dose and Weeks 1, 4, 8, 12, 20, and 26.

### Sample Analyses

#### Eculizumab Pharmacokinetics

Total (bound plus free) serum concentrations of eculizumab were measured by a validated enzyme-linked immunosorbent assay with a quantification range of 9.38–600 μg/ml.

#### Anti-AChR Antibody Titer

Anti-AChR antibody titers were determined by serological test in blood samples taken at screening and before eculizumab infusion at Weeks 12 and 26.

#### Free C5 Concentration

Concentrations of free C5 in serum (as a measure of target engagement) were measured by a validated ligand-binding assay using eculizumab for capturing free C5 and chemiluminescent detection [Meso Scale Discovery (MSD) platform; Meso Scale Diagnostics, Rockville, MD, USA]. The assay has a quantification range of 0.0274–20 μg/ml of C5. Serum free C5 <0.5 μg/mL represents complete inhibition of terminal complement (Alexion Pharmaceuticals, data on file).

#### *In vitro* Hemolytic Activity

Complement-mediated hemolytic activity in patient serum was measured *in vitro* by lysis of chicken red blood cells (cRBCs) using a validated semi-quantitative assay. cRBCs were sensitized with an anti-cRBC polyclonal antibody, incubated with serum, centrifuged, and the supernatant measured for hemoglobin released from cell lysis by absorbance (415 nm). Percent hemolysis was expressed relative to pooled normal human serum, as proof of the pharmacology of eculizumab. cRBC hemolysis <20% represents complete terminal complement inhibition (Alexion Pharmaceuticals, data on file).

#### Immunogenicity

To describe the presence or absence of an immune response to eculizumab, serum concentrations of ADAs against eculizumab were measured using a solution-phase bridging assay with electrochemiluminescent detection (MSD platform). The method used a tiered approach, with screening (sensitivity 6.26 ng/ml) and confirmatory competition steps to identify samples as positive or negative for the presence of ADAs to eculizumab. If ADAs to eculizumab were detected, the positive ADA samples were analyzed further for titer, and for the presence of neutralizing antibodies (NAbs) *via* a ligand-binding assay for inhibition of C5 binding with electrochemiluminescent detection (MSD platform).

### Population-Pharmacokinetic Model

One-compartment and two-compartment structural models were evaluated during model development. Body weight is known to be a predictive factor in the pharmacokinetics of therapeutic monoclonal antibodies. Therefore, the pharmacokinetic parameters clearance (CL), volume of distribution in the central compartment (V_1_), intercompartmental clearance (Q), and volume of distribution in the peripheral compartment (V_2_) were allometrically scaled by body weight as part of the structural model development.

Patients who experienced clinical deterioration (defined as MG crisis, substantial symptomatic worsening, or health in jeopardy if rescue therapy not given) during the study were permitted to receive rescue therapy, including plasmapheresis/plasma exchange (PLEX). To account for the increased clearance of eculizumab during PLEX, an effect of PLEX on CL was also included as part of the structural model development, rather than during the covariate search process.

As a starting point for the analysis, between-subject variability terms were described by an exponential equation. An attempt was made to define a full covariance matrix for the between-subject random effects when possible. Additive, exponential, and combined additive and exponential residual-error models were evaluated and the best residual-error model was selected based on minimum objective function value (OFV) and visual inspection of the plots of residuals vs. population predictions of eculizumab concentration.

The effect of covariates on the base pharmacokinetic model was analyzed: pairwise correlations of covariates and random effects from the base model were explored both graphically and using mixed-effects modeling. Covariates were identified using a stepwise covariate modeling procedure. This involved stepwise testing of covariate relationships in a forward inclusion [ΔOFV of 6.63, *p* < 0.01 for 1 degree of freedom (DF)] and backward exclusion (ΔOFV of 10.83, *p* < 0.001 for 1 DF) process. Continuous covariates were initially tested using a linear relationship; if found to be statistically significant they were then tested using a power function. Covariate–parameter relationships were predefined based on scientific interest, mechanistic plausibility, or prior knowledge, and the final model was constructed to avoid correlation or collinearity in predictors.

To assess whether dose alterations would be required in particular patient populations, a number of baseline covariates were evaluated, including demographic and clinical factors. A comprehensive list of covariates evaluated is included in Supplementary Methods.

Model development was guided by a number of criteria (see Supplementary Methods) with the aim of selecting the model that best described eculizumab disposition. A non-parametric bootstrap analysis was performed to evaluate performance and robustness of the final population-pharmacokinetic model.

### C5 Complement and Hemolytic Activity

The relationships between eculizumab concentration and free C5 and *in vitro* cRBC hemolytic activity were explored, with time-matched observed eculizumab concentrations as the exposure metric. Simulations were performed with the established pharmacokinetic and free C5 models in order to assess the eculizumab dosing regimen for free C5 response and to determine the target threshold concentration of eculizumab required to achieve complete terminal complement inhibition. Details of characterization of the relationship between hemolysis and C5 concentrations are provided in Supplementary Methods.

### Exposure–Response for Efficacy

The primary efficacy endpoint of the clinical study was the change in Myasthenia Gravis-Activities of Daily Living (MG-ADL) total score from baseline to Week 26 (worst-rank analysis of covariance). Secondary efficacy endpoints included changes from baseline to Week 26 in Quantitative Myasthenia Gravis (QMG) and Myasthenia Gravis Composite (MGC) total scores, and clinical deterioration as determined by the treating physician (10).

To explore the effect of eculizumab exposure on MG-ADL, QMG, and MGC scores and clinical deterioration, individual steady-state area under the concentration–time curve (AUC) values within the 2-week dosing interval were derived from *post hoc* exposure estimates and were used as the exposure metric.

### Exposure–Response for Safety

Treatment-emergent AEs of special interest—defined as infections (meningococcal infections, *Aspergillus* infections, other serious infections, or sepsis), infusion reactions, serious cutaneous adverse reactions, cardiac disorders, and angioedema—and treatment-emergent AEs that occurred in ≥ 5% of the eculizumab-treated patients were evaluated by treatment and by eculizumab exposure quartile. Individual steady-state eculizumab AUC values were derived from *post hoc* exposure estimates and were used as the exposure metric.

## Results

The full analysis set comprised 62 adult patients who received eculizumab treatment during the REGAIN study, including three Japanese patients. Pharmacokinetic parameters were available for all 62 patients. Patient characteristics as well as efficacy and safety results have been reported previously (10). In brief, the mean [standard deviation (SD)] baseline characteristics of the full analysis set were: age at diagnosis of MG 38.0 (17.8) years; duration of MG before screening 9.9 (8.1) years; age at first study dose of eculizumab 47.5 (15.7) years; body weight 87.7 (28.2) kg; BMI 31.4 (9.0) kg/m^2^; eGFR 88.7 (23.3) ml/min/1.73 m^2^; white blood cell count 8.6 (3.8) × 10^9^/L; and albumin 43.1 (3.1) g/L. The mean (SD) anti-AChR antibody titer was 94.3 (172.4) nmol/L (median 11.4 nmol/L, range 0–892 nmol/L). Of the 62 patients in the analysis set, 53 patients (85%) were White and three (5%) were Asian (Japanese); there were no Black/African American patients and six (10%) were of multiple, unknown, or other ethnicity. Eleven PLEX events were recorded for four patients during the study.

### Pharmacokinetics

#### Serum Eculizumab Concentrations

**Figure 1:**
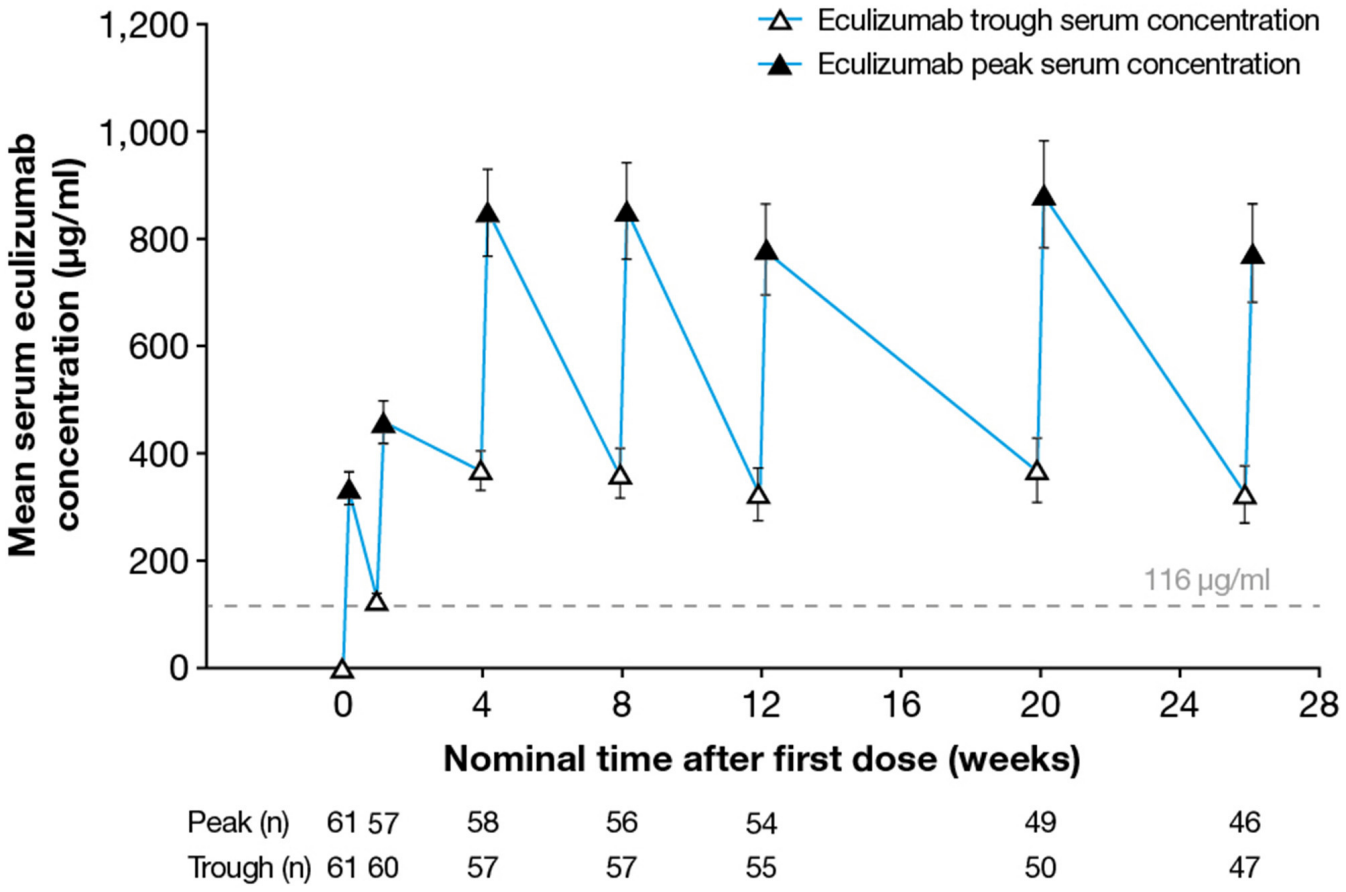
Serum eculizumab concentrations during the study. Mean (95% CI) trough and peak serum eculizumab concentrations in patients who received eculizumab in the REGAIN study; eculizumab concentrations below the lower limit of quantification (9.38 μg/ml) were analyzed as 0 μg/ml. Eculizumab concentrations above 116 μg/ml (dashed line) indicate sufficient concentration to achieve complete complement inhibition (see Figure 3). CI, confidence interval.

Following implementation of the eculizumab 900/1,200 mg dosing regimen, steady-state serum eculizumab concentrations were achieved by Week 4 (as measured in the sample taken before the fifth dose was administered) and were sustained throughout the 26-week treatment period (Figure 1). Between Weeks 4 and 26 of eculizumab treatment, the median peak eculizumab concentration (C_max_) by visit ranged from 742 to 920 μg/ml, with the lower bound of the 90% confidence interval (CI) ranging from 318 to 430 μg/ml and the upper bound of the 90% CI ranging from 1,230 to 1,480 μg/ml. The median trough eculizumab concentration (C_trough_) ranged from 336 to 386 μg/ml, with 90% CI boundaries of 52.8–170 μg/ml (lower) to 558–679 μg/ml (upper).

#### Population-Pharmacokinetic Model

The model that best described the eculizumab pharmacokinetic data was a two-compartment population-pharmacokinetic model with first-order elimination. In this model, CL, V_1_, and V_2_ were estimated with good precision, with percent relative standard error (%RSE) below 10%. Random effects were included for CL and V_1_ and a correlation term was estimated between the two. A proportional-error model was sufficient to describe the residual variability of the data. All parameter estimates were physiologically plausible and were estimated with good precision (%RSE <30%). Parameter estimates for the final population-pharmacokinetic model are provided in Supplementary Table 1.

All pharmacokinetic parameters were allometrically scaled by body weight; in addition, PLEX events were modeled to account for a temporary increase in eculizumab CL during the PLEX administration period. The covariate analysis did not identify any other factors that significantly affected eculizumab exposure. None of the other covariates tested (see Supplementary Methods) resulted in a statistically significant improvement of the model according to the predefined criteria, and were therefore not included in the final population-pharmacokinetic model.

There was no noticeable bias in goodness-of-fit plots of observed vs. predicted population or individual eculizumab concentrations, nor of conditional weighted residual error vs. population prediction or time, indicating that eculizumab exposure was characterized appropriately. Visual predictive checks of the mean and 90% prediction interval overlaid with the observed median and 5th and 95th percentile showed good overlap of simulated and observed data, indicating that the model described the observed data well (Supplementary Figure 1).

The final population model successfully described the eculizumab concentration time course and was used to derive pharmacokinetic exposure in patients with refractory gMG. Bayesian estimates of the pharmacokinetic parameters are summarized in Table 1; the mean (SD) terminal half-life of eculizumab was estimated to be 436 (152) h [18.2 (6.3) days], and the mean (SD) clearance was estimated to be 0.0106 (0.00783) L/h.

**Table 1:** Summary of estimated pharmacokinetic parameters.

**Parameter**	**Mean**	**SD**	**Median**	**Minimum**	**Maximum**
CL (L/h)	0.0106	0.00783	0.00785	0.00362	0.0448
V_1_ (L)	2.54	0.758	2.44	1.32	5.25
V_2_ (L)	2.73	0.554	2.61	1.76	4.26
Q (L/h)	0.251	0.107	0.218	0.0956	0.605
Observed C_max_ (μg/ml)	939	353	912	265	1,640
C_max, ss_ (μg/ml)	845	295	878	253	1,580
C_trough, ss_ (μg/ml)	348	180	348	29.6	831
Terminal half-life (h)	436	152	453	136	952
AUC_ss_ (μg·h/ml)	154,000	68,600	153,000	26,800	331,000

*Data are summaries of the individual Bayesian estimates. AUC_ss_, area under the concentration–time curve at steady state; CL, clearance; C_max_, peak concentration; C_trough_, trough concentration; Q, intercompartmental clearance; SD, standard deviation; ss, steady state; V_1_, volume of distribution in the central compartment; V_2_, volume of distribution in the peripheral compartment*.

#### Eculizumab Pharmacokinetics in Japanese Patients

Although a formal pharmacokinetic analysis focusing on Japanese patients was not possible due to the small number of Japanese patients (*n* = 3) in the study, no apparent differences in eculizumab pharmacokinetics were observed between Japanese and non-Japanese patients (Supplementary Table 2). With the exception of intercompartmental clearance Q for one Japanese patient, all pharmacokinetic parameters from the three Japanese patients were within the 5th–95th percentile range for non-Japanese patients. Any apparent trends toward higher eculizumab serum concentrations (peak and trough) in Japanese patients compared with the non-Japanese patients were likely due to the lower body weight of the Japanese patients.

### Immunogenicity

There were no detectable ADAs or NAbs in eculizumab-treated patients. A single positive ADA result occurred in a sample taken before eculizumab treatment; the NAb result from the sample was negative.

### Pharmacodynamics

#### Serum Free C5 Concentrations

**Figure 2:**
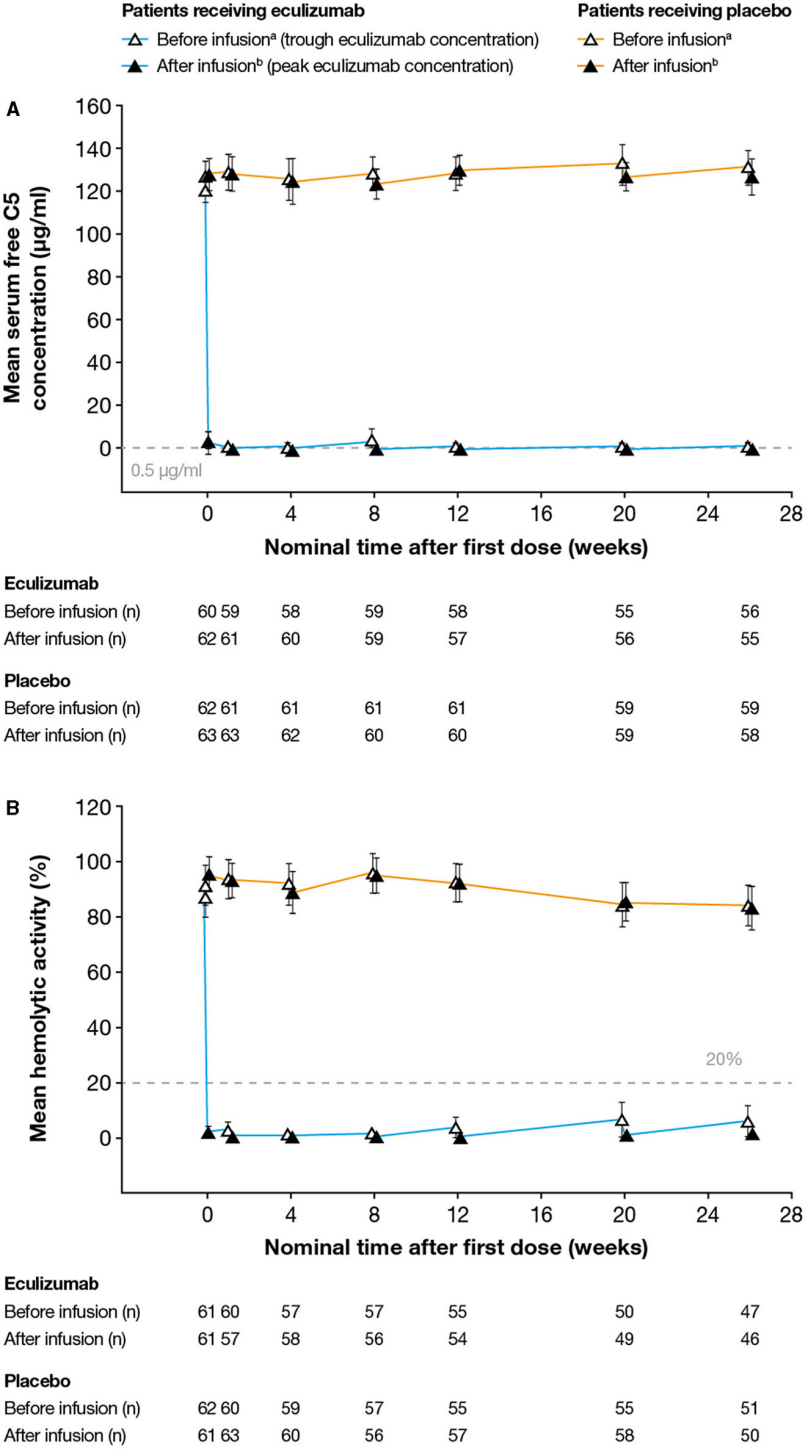
Serum free C5 concentrations and complement-mediated hemolytic activity in serum during the study. **(A)** Mean (95% CI) serum free C5 concentrations. Free C5 concentrations below the lower limit of quantification (0.0274 μg/ml) were analyzed as 0.0137 μg/ml. Free C5 concentrations below 0.5 μg/ml (dashed line) indicate complete terminal complement inhibition. **(B)** Mean (95% CI) percent *in vitro* complement-mediated hemolytic activity of serum samples. Hemolysis values above 20% (dashed line) indicate incomplete inhibition of hemolysis. For both analyses, samples were taken before and after eculizumab infusion (i.e., at eculizumab serum trough and peak concentrations, respectively); samples taken before and after infusion in patients receiving placebo are shown for comparison. ^a^Samples taken 5–90 min before infusion; ^b^Samples taken 60 min after the completion of infusion; ^c^Day 1. The numbers reported below the graphs are the numbers of patients for whom samples were tested at that timepoint. C5, complement protein 5; CI, confidence interval.

Serum free C5 concentrations <0.5 μg/ml were observed in 744/760 (97.9%) of post-baseline samples from eculizumab-treated subjects. Following the first dose of eculizumab treatment, complete inhibition of C5 (serum free C5 concentration <0.5 μg/ml) at the time of trough eculizumab concentrations was observed for 57/62 eculizumab-treated patients (92%) (including all three Japanese patients) at all visits throughout the study (Figure 2A). In the remaining patients, complete inhibition was seen at C_trough_ at the majority of visits during the maintenance phase of treatment. Complete inhibition was seen at peak eculizumab concentration for all patients at all visits.

#### Hemolysis

Complete inhibition of *in vitro* complement-mediated hemolytic activity (i.e., < 20% hemolysis in the cRBC hemolytic assay) was observed at the time of trough concentration in all samples taken from 54/62 eculizumab-treated patients (87%) (Figure 2B). Samples taken from the remaining patients at the time of eculizumab C_trough_ at the majority of visits in the maintenance phase of the study showed complete inhibition of *in vitro* hemolytic activity. Complete inhibition of hemolytic activity was seen at peak eculizumab concentration for all patients at all visits. Full inhibition of hemolytic activity was seen in all samples from all three Japanese patients throughout the study.

#### Population-Pharmacokinetic/Pharmacodynamic Modeling of Serum Free C5 and Hemolytic Activity

The relationship between eculizumab concentration and both serum free C5 and hemolytic activity was best described by an inhibitory sigmoid E_max_ model. Visual predictive checks of the final free C5 and hemolysis models indicated that they described the observed data reasonably well (Supplementary Figures 2, 3, respectively). The modeled eculizumab exposure–response profile for serum free C5 is shown in Supplementary Figure 4: the eculizumab median inhibitory concentration (IC_50_) estimate of 33.1 μg/ml from the model is consistent with the binding kinetics between eculizumab and free C5 (i.e., one molecule of eculizumab would bind two molecules of free C5).

**Figure 3:**
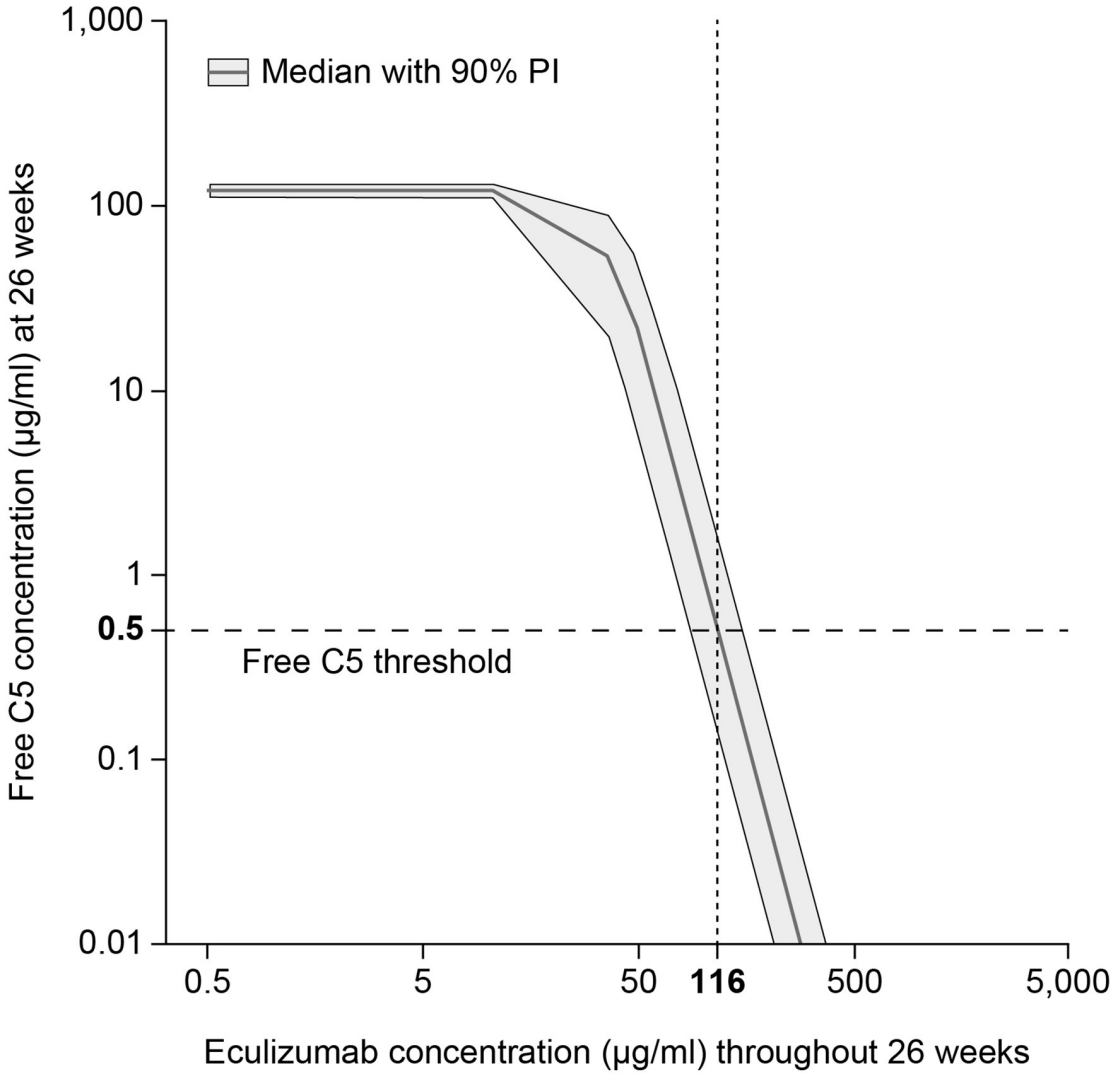
Target eculizumab concentration threshold providing complete complement inhibition (serum free C5 ≤ 0.5 μg/ml). Simulated exposure–response profile of free C5 concentration vs. eculizumab concentration, summarized using median response (gray solid line) and showing the 90% PI (gray shading). Using the threshold value 0.5 μg/ml, which is predicted to produce 20% hemolysis, the target concentration of eculizumab was identified as 116 μg/ml. C5, complement protein 5; PI, prediction interval.

The pharmacokinetic/pharmacodynamic modeling and simulation of free C5 concentration vs. eculizumab concentration identified the target serum concentration of eculizumab required to achieve complete terminal complement inhibition (i.e., serum free C5 concentration < 0.5 μg/ml) as 116 μg/ml (Figure 3).

### Exploratory Exposure–Response Analysis

Efficacy results have been reported previously (10).

#### Anti-AChR Antibodies

Anti-AChR antibody titers were highly variable; there was no relationship between eculizumab exposure and anti-AChR antibody titers.

#### Efficacy: MG-ADL, QMG, and MGC Scores

**Figure 4:**
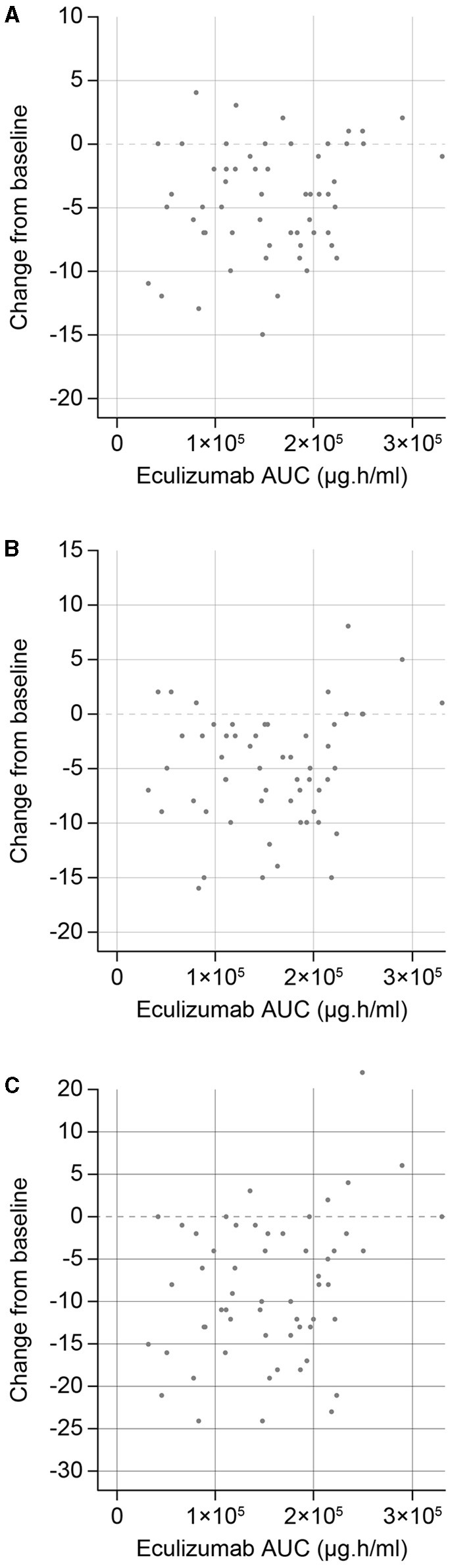
Eculizumab exposure–response efficacy profiles for changes in **(A)** MG-ADL, **(B)** QMG, and **(C)** MGC total scores from baseline to Week 26. AUC, steady-state area under the concentration–time curve within the 2-week dosing interval; MG-ADL, Myasthenia Gravis-Activities of Daily Living; MGC, Myasthenia Gravis Composite; QMG, Quantitative Myasthenia Gravis evaluation.

As reported previously, sensitivity analyses demonstrated that eculizumab treatment was associated with statistically significant improvements in MG-ADL, QMG, and MGC scores compared with placebo (10). In the current analysis, exploratory exposure–response plots were generated for the change from baseline of efficacy endpoints of interest (MG-ADL, QMG, and MGC total scores) (Figure 4). It was concluded that no further exposure–response modeling was needed, as there was no consistent evidence of increased efficacy with higher levels of eculizumab exposure (stratified AUC) at the eculizumab concentrations achieved during the study. This suggests that the efficacy reached a maximum plateau, consistent with use of a therapeutic dose that achieves full inhibition of terminal complement activation.

#### Efficacy: Clinical Deterioration

There was no evidence of a relationship between eculizumab exposure (stratified AUC) and the occurrence of clinical deterioration within the observed eculizumab exposure range. A total of 7/62 eculizumab-treated patients (11%) experienced one or more MG-related clinical deterioration events during or immediately after the 26-week treatment period. The incidence of first clinical deterioration was similar in the low [below median eculizumab AUC; 3/31 (10%)] and high [above or equal to median eculizumab AUC; 4/31 (13%)] eculizumab-exposure groups. For comparison, 16/63 patients (25%) who received placebo experienced one or more events of clinical deterioration.

#### Safety

The incidence and type of AEs was similar between placebo- and eculizumab-treated patients. No trends in the occurrence of AEs were observed with increasing eculizumab exposure, comparing patients treated with eculizumab stratified in terms of the steady-state eculizumab AUC quartiles.

## Discussion

Results of this pharmacokinetic/pharmacodynamic analysis provide evidence-based confirmation of the recommended dosing regimen for intravenous eculizumab in adult patients with anti-AChR antibody-positive refractory gMG, which comprises 900 mg weekly for the first four doses, followed by 1,200 mg 1 week later and every 2 weeks thereafter. A two-compartment population-pharmacokinetic model with first-order elimination best describes the observed eculizumab pharmacokinetic profile following intravenous infusion in patients with anti-AChR antibody-positive gMG. The model confirmed that eculizumab clearance is affected by body weight and PLEX. Although some pharmacokinetic parameters were affected by body weight, the data suggest that no dose adjustments are needed in overweight patients with gMG. None of the other patient-related factors examined significantly affected eculizumab pharmacokinetics in these patients.

The final model reflected the increased clearance and reduced elimination half-life of eculizumab in patients receiving PLEX or fresh frozen plasma infusions (20). Fresh frozen plasma infusion would also likely introduce a bolus of complement proteins (21). A supplemental dose of eculizumab is recommended after PLEX administration to rectify a possible dilution effect on eculizumab (13, 14). In REGAIN, patients were excluded if they had received PLEX within the 4 weeks before study initiation in order to reduce the potential for such effects. However, patients who experienced clinical deterioration during the study could receive plasmapheresis or PLEX as rescue therapy. If PLEX was administered on a day of regularly scheduled eculizumab administration, patients received the regularly scheduled dose within 60 min after the end of the PLEX session. If PLEX was administered on any other day, eculizumab 600 mg was given within 60 min after the end of each PLEX session.

Japanese and non-Japanese patients appeared to have similar eculizumab pharmacokinetic profiles, and no differences were observed with regard to complement inhibition; however, it should be noted that data were only available for three Japanese patients. Nevertheless, based on the totality of the data, it would appear that eculizumab dose adjustment and dose-regimen changes are not required in specific populations (e.g., Japanese patients) or based on renal or hepatic function.

This report highlights the model-based analyses, information, and variability that are factored into determining a dose regimen, and the importance of adherence to the recommended dosing interval for eculizumab. An alternative to the per-protocol dose regimen presented here is therapeutic drug monitoring, which is a real-time method for ensuring that patients receive an effective dose where safety is a concern or drug exposure assessment is complicated. However, safety is not a concern with eculizumab, and exposure is well-characterized using a population-pharmacokinetic model. The terminal elimination half-life of 18.2 days in patients with gMG confers a convenient interval between doses and provides some allowance for day-to-day fluctuations in C5 concentrations [e.g., as a result of complement-activating events, such as infection, surgery (including thymectomy), or pregnancy, which may increase C5 concentrations], and for slight variation in dosing schedules (± 2 days), without risk of incomplete terminal complement inhibition (22, 23). Complete terminal complement inhibition (defined as serum free C5 < 0.5 μg/ml or *in vitro* cRBC hemolysis < 20%; Alexion Pharmaceuticals, data on file) was achieved by the end of infusion of the first dose and was sustained throughout the 6-month treatment period in over 90% of patients. This supports terminal complement inhibition as the mechanism by which eculizumab exerts its therapeutic effects with rapid onset in patients with gMG (2, 20). Exposure–response modeling established that a target serum eculizumab concentration of 116 μg/ml achieved complete complement inhibition, as indicated by a serum concentration of free C5 < 0.5 μg/ml. Patients maintained a median trough serum eculizumab concentration above this threshold from Week 1 of treatment to the end of the study.

Results of exposure–response analyses were compatible with the use of a therapeutic dosing regimen that achieves full inhibition of terminal complement activation. Clinical efficacy (assessed by MG-ADL, QMG, or MGC total scores and the incidence of clinical deterioration) was consistent across the full exposure range, indicating that the maximum therapeutic effect had been achieved. No relationship was seen between eculizumab exposure and anti-AChR antibody titers; this is as expected based on the mechanism of action of eculizumab, which targets C5 in the complement cascade, distinct from targeting anti-AChR antibody. To date, there are no published data to suggest that anti-AChR antibody titer, which is highly variable, correlates with disease activity in patients with anti-AChR antibody-positive gMG.

Consistent with the very low rate of samples testing positive for ADAs or NAbs in eculizumab studies in other indications (24–26), there was no evidence of ADAs or NAbs in eculizumab-treated patients in this study (the ADA titer was very low in the single positive ADA sample, which was taken before eculizumab treatment and was most likely a false-positive finding resulting from the stringency of the criteria). This reflects the design of the antibody (IgG2/IgG4) to minimize immunogenicity (2), and indicates that sustained efficacy with long-term use of eculizumab can be expected (27).

No safety trends were observed with increased serum eculizumab concentrations in this analysis. Results of the REGAIN study showed eculizumab to be generally well-tolerated in patients with gMG (10). The overall incidence of treatment-emergent AEs during the double-blind treatment phase was similar in the eculizumab and placebo groups (86 vs. 89%, respectively). The types of AEs were also similar in the treatment groups; most were mild to moderate in severity and considered unrelated to the study drug.

The terminal complement pathway plays a key role in clearing the bacterium *Neisseria meningitidis* (28); use of complement inhibitors may therefore increase the risk of invasive meningococcal disease. However, vaccination-based risk-mitigation strategies are available, with *N. meningitidis* serogroups ACWY vaccination and, where available, *N. meningitidis* serogroup B vaccination, recommended before initiating eculizumab therapy (13, 14). No meningococcal infections were reported during the double-blind phase of the REGAIN study, in which all randomized patients were required to have been vaccinated against *N. meningitidis* (10). One case of meningococcal infection occurred during the open-label extension study; this resolved with antibiotic treatment, after which the patient resumed treatment with eculizumab (11). In the largest safety dataset analyzed to date, representing almost 10 years of post-marketing pharmacovigilance surveillance worldwide of eculizumab in the treatment of paroxysmal nocturnal hemoglobinuria (PNH) and atypical hemolytic uremic syndrome (aHUS) [cumulative exposure, 28,518 person-years (PY)], the overall incidence of meningococcal disease was 0.25/100 PY and the associated mortality rate was 0.03/100 PY (29). The rate of meningococcal infection decreased over time from 0.57/100 PY in 2007 to 0.16/100 PY in 2016 (29); this is consistent with the decline in meningococcal infections in the general population during this period following introduction of the Men ACWY meningococcal vaccine from 2005 onwards (30). A total of 16 cases of meningococcal infection were identified by the Centers for Disease Control and Prevention in eculizumab-treated patients in the US between 2008 and 2016 (31).

While published data are lacking regarding the potential for drug–drug interactions with other medications likely to be used in patients with gMG (20), the current analysis did assess some potential drug–drug interactions through covariate testing of concomitant medications. Patients were permitted to remain on oral immunosuppressive medications (including corticosteroids, azathioprine, and mycophenolate mofetil) and cholinesterase inhibitors in the REGAIN study (10). In the present analysis, no evidence was found that any of these concomitant medications affected the pharmacokinetic profile of eculizumab, suggesting a lack of interaction. This is expected for a monoclonal antibody such as eculizumab, which is unlikely to be metabolized by cytochrome P450 or other metabolic enzymes.

Our findings for MG are consistent with those of the accompanying pharmacometric analysis of eculizumab in patients with aquaporin-4 IgG-positive NMOSD (26). Clinical efficacy and safety of eculizumab have also been reported in aquaporin-4 IgG-positive NMOSD (32, 33), PNH (34, 35), and aHUS (36, 37)—clinical conditions in which eculizumab is also effective *via* its mechanism of inhibition of terminal complement activation.

Ravulizumab is another complement-targeting drug that has now been approved for treatment of PNH and aHUS in the USA (38) and Europe (39) and which maintains a sustained inhibition of complement for 8 weeks following intravenous administration (40, 41). The ravulizumab dose regimen has been optimized to account for a patient's body weight, minimizing potential overexposure and covering 97.5% of the patient population. Ravulizumab is currently in development for the treatment of MG. Other complement-targeting drugs are also in development for the treatment of MG, including a small-molecule C5 inhibitor that has been shown to achieve near-complete complement blocking in patients with MG (42).

A strength of this analysis is the relatively large sample of patients included, particularly considering that gMG is a rare disease. Potential limitations are the low number of Asian patients included and the lack of patients of other ethnicities, which limits the ability to compare different ethnic groups.

In summary, this rigorous quantitative pharmacokinetic model-based analysis of exposure–response provides evidence-based validation of the recommended dosing regimen for intravenous eculizumab in adult patients with anti-AChR antibody-positive refractory gMG. Rapid and complete inhibition of terminal complement activation was shown to be achieved after the first dose, with the 2-week maintenance dosing interval providing trough serum eculizumab concentrations above the threshold required to achieve complete terminal complement inhibition and sustained clinical benefit.

## Data Availability Statement

The datasets presented in this article are not readily available; Alexion will consider requests for disclosure of clinical study participant-level data provided that participant privacy is assured through methods like data de-identification, pseudonymization, or anonymization (as required by applicable law), and if such disclosure was included in the relevant study informed consent form or similar documentation. Qualified academic investigators may request participant-level clinical data and supporting documents (statistical analysis plan and protocol) pertaining to Alexion-sponsored studies. Further details regarding data availability and instructions for requesting information are available in the Alexion Clinical Trials Disclosure and Transparency Policy at https://alexion.com/our-research/research-and-development. Requests to access the datasets should be directed to https://alexion.com/contact-alexion/medical-information.

## Ethics Statement

The studies involving human participants were reviewed and approved by independent Ethics Committees or institutional review boards at the 76 centers that participated in the study (see Supplementary Material for full list). The patients/participants provided their written informed consent to participate in this study.

## Author Contributions

JM: pharmacometric strategy, pharmacokinetic/ pharmacodynamic simulations, report writing, critical review, editing, and approval of manuscript. XG: protocol design, analysis planning and strategy, data analyses and interpretation, report writing, critical review, editing, and approval of manuscript. HJK: data analysis, interpretation, report writing, critical review, editing, and approval of manuscript. FB: data analysis, interpretation, critical review, editing, and approval of manuscript. RP: bioanalyses, critical review, editing, and approval of manuscript. All authors contributed to the article and approved the submitted version.

## Funding

Editorial assistance was provided by Duncan Porter and Jennifer Coward of Piper Medical Communications, funded by Alexion Pharmaceuticals Inc.

## Conflict of Interest

JM and RP are employed by Alexion Pharmaceuticals, Inc.; XG was employed by Alexion Pharmaceuticals, Inc. at the time the work described in this paper was undertaken; HJK and FB are employed by Certara Strategic Consulting, which received funding from Alexion Pharmaceuticals, Inc.

## Publisher's Note

All claims expressed in this article are solely those of the authors and do not necessarily represent those of their affiliated organizations, or those of the publisher, the editors and the reviewers. Any product that may be evaluated in this article, or claim that may be made by its manufacturer, is not guaranteed or endorsed by the publisher.
