# Supplementary material for: Eculizumab Pharmacokinetics and Pharmacodynamics in Patients With Generalized Myasthenia Gravis
Source: Front Neurol. 2021 Nov 2;12:696385. doi: 10.3389/fneur.2021.696385 (PMC8594444; doi:10.3389/fneur.2021.696385)
Supplement: Supplementary file 1 [file Data_Sheet_1.docx]

Supplementary Material

# Supplementary Methods

## Software

SAS (version 9.4; SAS Institute Inc., Cary, NC, USA) software was used to perform dataset assembly and pharmacokinetic/pharmacodynamic analysis of hemolysis. The population-pharmacokinetic analysis was performed using a qualified installation of NONMEM (version 7.2; ICON Development Solutions, Hanover, MD, USA) with an Intel FORTRAN compiler (version 12). Perl-speaks-NONMEM (PsN; version 4.2.0) was employed as a user interface to NONMEM. The R-language (version 3.3.1) (1) was used for post-processing of modeling results.

## Dataset

Source data were provided from a locked database, and included: dosing information, demographic information, pharmacokinetic and pharmacodynamic measurement information, and laboratory measurements.

Eculizumab serum concentrations reported as below the limit of quantification (BLQ) were excluded from the population-pharmacokinetic analysis since the number of BLQ samples was low (n=69 and 7.2% of total observations), with only two post-dose samples reported as BLQ (67 out of the 69 BLQ values were prior to eculizumab administration). However, the records were maintained in the analysis dataset and flagged to ensure complete traceability.

Missing covariates were imputed using study-specific median values.

Free C5 and hemolysis observations without a time-matched eculizumab concentration were excluded from the exposure–response modeling.

For exposure–response modeling, eculizumab concentrations were set to 0 for pre-treatment BLQ samples and to half the lower limit of quantification (LLOQ) for concentrations that were BLQ during treatment. Free C5 concentrations that were BLQ were set to 1/2*LLOQ.

## Pharmacokinetic Model Development

One- and two-compartment pharmacokinetic structural models were developed and evaluated based on minimum objective function value (OFV), while also considering change in degrees of freedom, as well as visual inspection of conditional weighted residuals (CWRES) vs time plots. Preference was given to the simplest model that would describe the data best. The effects of body weight on pharmacokinetic parameters [clearance (CL), volume of distribution in the central compartment (V_1_), intercompartmental clearance (Q), and volume of distribution in the peripheral compartment (V_2_)] and of plasma exchange on CL were included in the base model.

### Between-Subject Variability and Residual Error

As a starting point for the analysis, between-subject variability terms were described by an exponential equation:

*Equation 1*

Where *P_i_* is the estimated parameter value for individual *i* and θ is the typical population value of the parameter. η*_pi_* are individual-specific between-subject random effects for individual i and parameter P, and are assumed to be distributed: η ~N (0, Ω^2^). An attempt was made to define a full covariance matrix for the between-subject random effects (Ω) where possible.

Additive (Equation 2), exponential (Equation 3), and combined additive and exponential (Equation 4) residual-error models were evaluated, and the best residual-error model was selected based on minimum OFV and visual inspection of residuals vs population predictions (PREDs) of Y (concentrations for pharmacokinetic model) plot:

*Equation 2*

*Equation 3*

*Equation 4*

Where, Yij is the jth measured observation in individual i. Yˆij is the jth model-predicted value in individual i. ε_ADDij and ε_PROPij are additive and proportional residual random errors, respectively, for individual i and measurement j and are each assumed to be independently and identically distributed.

### Covariate Analysis

A covariate search was performed using the stepwise covariate model search built in the PsN program. Both forward selection and backward elimination were performed, and the default p-values of p<0.01 (1 degree of freedom) for forward inclusion and p<0.001 (1 degree of freedom) for backward elimination were used to determine statistical significance.

### Covariates Evaluated in the Population-Pharmacokinetic Model

Covariates evaluated were: age, sex, race, ethnicity (Japanese descent), body weight, height, and body mass index; baseline estimated glomerular filtration rate and serum creatinine; baseline measurements of alanine transaminase, aspartate transaminase, alkaline phosphatase, albumin, and bilirubin; white blood cell count and anti-acetylcholine receptor antibody titer at baseline; baseline Myasthenia Gravis-Activities of Daily Living and Quantitative Myasthenia Gravis scores; Myasthenia Gravis Foundation of America classification and therapy status at screening; thymectomy status; use of myasthenia gravis medications, including immunosuppressants; use of other immunosuppressants; use of systemic antibacterial medication; use of intravenous immunoglobulin; use of anti-anemic medication; and use of a cholinesterase inhibitor.

### Criteria for Model Acceptance

Model development was guided by a number of criteria with the aim of selecting the model that best described eculizumab disposition. These included successful numeric convergence, obtaining a successful covariance step, acceptably low condition number, decrease in OFV, goodness-of-fit plots showing good agreement of model predictions to observed data, an acceptable visual predictive check, acceptable precision of estimated parameters (preferably a relative standard error of estimation (RSE) no higher than 50% of the parameter estimate) with physiologically plausible estimated values, and no significant bias in the random-effects estimates.

### Model Assessment Process

Model development was assessed using standard diagnostics based on model outputs. Parameter estimates were typically reported as a point estimate with a 95% confidence interval (CI), derived from asymptotic-normal standard errors obtained from the covariance matrix of the estimates (NONMEM $COV step).

Assessment of model goodness of fit was based on multiple criteria:

- Visual inspection of diagnostic plots (i.e., observed vs predicted value, residual/weighted residual vs predicted value or time, and histograms of individual random effects). Note that tables and plots used the general term CWRES to reflect a conditional weighted residual. The appropriate weighted residual was presented for all diagnostics, depending on the estimation methods used. In the case of all models, conditional estimation was implemented, but η—ε interaction was only used where the prediction was involved in the weighting scheme. In those cases, the NONMEM CWRES was presented in plots.
- Plausibility of parameter estimates.
- Precision/uncertainties in parameter estimates.
- Minimum OFV and number of estimated parameters.
- Successful convergence of the minimization routine with at least three significant digits in parameter estimates.

The final population-pharmacokinetic models were evaluated using a simulation-based predictive check method in addition to standard diagnostic plots. This method is derived from the posterior predictive check methods, but assumes that parameter uncertainty is negligible relative to between-subject and residual variance. Five hundred Monte Carlo simulation replicates of the original dataset were generated using the final population-pharmacokinetic model. For the pharmacokinetic predictive checks, observations of predicted values BLQ were excluded. Distributions of a characteristic of the simulated data were compared with the distribution of the same characteristic in the observed dataset, using visual predictive checks. Any problems evident by visual inspection during visual predictive checks were investigated and further model development was conducted as necessary.

A bootstrap procedure was carried out to obtain parameter uncertainty estimates and to identify influential observations. In the bootstrap resampling technique, bootstrap replicates were generated by sampling randomly from the original data set with replacement. One thousand replicate data sets were obtained using the bootstrap option in the PsN software package. Parameter estimates for each of the resampled data sets were obtained by fitting the final model using NONMEM, and non-parametric CIs of the parameter estimates were derived.

### Initial Development of Base Two-Compartment Model

The starting point for model development was a one-compartment pharmacokinetic model developed for an earlier Phase 2 crossover study of eculizumab in patients with generalized myasthenia gravis (Study C08-001; ClinicalTrials.gov Identifier: NCT00727194). In this model, clearance and volume of distribution were allometrically scaled by body weight. The one-compartment model included a PLEX effect on eculizumab clearance to account for changes in clearance due to PLEX procedures. Visual inspection of the conditional weighted residual (CWRES)–time profile showed a clear bias in the description of trough concentration data collected during the induction phase. A two-compartment model was found to address the bias and to provide a more adequate description of eculizumab exposure, with improved goodness of fit (Supplementary Figure 5).

### Characterization of C5–Hemolysis Relationship Using In Vitro cRBC Hemolysis and Free C5 Data

A serum free C5 vs hemolysis correlation experiment was performed at Alexion Pharmaceuticals using a validated assay that measures the level of hemoglobin release from sensitized chicken red blood cells (cRBCs) as a result of lysis by the human terminal complement complex (C5b-9) deposited on the cell surfaces (2).

Using the NONMEM software version 7.2 (ICON Development Solutions, Hanover, MD, USA) a pharmacodynamic/pharmacodynamic analysis was performed by developing a nonlinear model to characterize the relationship between cRBC hemolysis and free C5. Supplementary Figure 6 shows the experimental data and the final model fit through the data points.

Using the pharmacodynamic/pharmacodynamic model and final parameter estimates, prediction of free C5 can be made at specified mean (90% CI) hemolysis levels. Using a rule that the lower bound of the 90% CI is greater than zero, free C5 was predicted to be 0.309 μg/mL (Supplementary Table 3). This free C5 concentration of 0.309 μg/mL was identified as a free C5 concentration, above which a positive hemolysis response, i.e., >0%, can be predicted by the model. For a hemolysis of 20%, the predicted free C5 concentration was 0.51 μg/mL.

Thus, it is possible to set a free C5 threshold (~0.5 µg/mL) below which hemolysis is expected to be <20%.

## References

1. R Core Team. R: A language and environment for statistical computing. Vienna: R Foundation for Statistical Computing (2016). <https://www.r-project.org>

2. Alexion Pharmaceuticals, Inc. CPR-0013.00 (internal report). Characterization of C5-hemolysis relationship using in vitro cRBC hemolysis and free C5 data. 04 Nov 2016.

# Supplementary Results

**SUPPLEMENTARY TABLE 1 |** Parameter estimates of final population-pharmacokinetic model.

|  |  |  | **Bootstrap** | |
| --- | --- | --- | --- | --- |
| **Parameter** | **Estimate** | **%RSE** | **Median** | **95% CI** |
| CL (L/h) | 0.0071 | 1.16 | 0.00703 | 0.0064, 0.0076 |
| V_1_ (L) | 2.15 | 9.14 | 2.16203 | 1.78, 2.48 |
| Q (L/h) | 0.19 | 27.01 | 0.18 | 0.025, 0.39 |
| V_2_ (L) | 2.55 | 8.31 | 2.52 | 1.89, 3.03 |
| WT effect on CL/Q | 1.28 | 11.94 | 1.28 | 0.97, 1.67 |
| WT effect on V_1_/V_2_ | 0.63 | 11.29 | 0.63 | 0.46, 0.81 |
| PLEX effect on CL | 11.59 | 12.38 | 11.62 | 7.57, 720.72 |
| BSV CL (CV %) | 38.01 | 9.26 | 37.30 | 29.41, 44.65 |
| BSV V_1_ (CV %) | 22.53 | 13.51 | 21.77 | 16.29, 29.63 |
| Correlation CL–V_1_ | 0.40 | 18.43 | 0.39 | 0.02, 0.5 |
| Proportional error (%) | 15.36 | 2.87 | 15.30 | 13.57, 16.99 |

BSV, between-subject variability; CI, confidence interval; CL, clearance; CV, coefficient of variation; PLEX, plasma exchange or plasmapheresis; Q, intercompartmental clearance; RSE, relative standard error; V_1_, volume of distribution in the central compartment; V_2_, volume of distribution in the peripheral compartment; WT, weight.

**SUPPLEMENTARY TABLE 2 |** Pharmacokinetic parameters for Japanese and non-Japanese patients.

|  | **Non-Japanese patients (n=59)** | | | | | **Japanese patients (n=3)** | | |
| --- | --- | --- | --- | --- | --- | --- | --- | --- |
| **Parameter** | **Median** | **5P** | **25P** | **75P** | **95P** | **Patient A BW 60.1 kg** | **Patient B BW 51.2 kg** | **Patient C BW 63.4 kg** |
| CL (L/h) | 0.008 | 0.0048 | 0.0059 | 0.0127 | 0.0265 | 0.0051 | 0.0054 | 0.0078 |
| V_1_ (L) | 2.49 | 1.57 | 1.96 | 3.09 | 3.95 | 2.19 | 1.7 | 2.09 |
| V_2_ (L) | 2.62 | 2.02 | 2.37 | 3.14 | 3.63 | 2.18 | 1.97 | 2.25 |
| Q (L/h) | 0.22 | 0.128 | 0.178 | 0.32 | 0.433 | 0.149 | 0.121 | 0.16 |
| C_max,ss_ (µg/mL) | 785 | 397 | 620 | 1050 | 1270 | 1100 | 1190 | 890 |
| C_trough,ss_ (µg/mL) | 347 | 64.5 | 194 | 472 | 605 | 569 | 503 | 330 |
| Terminal half-life (h) | 451 | 177 | 338 | 513 | 616 | 560 | 473 | 390 |
| AUC_ss_ (µg·h/mL) | 151000 | 45300 | 94900 | 203000 | 250000 | 236000 | 220000 | 153000 |

5P, 25P, 75P, and 95P, 5th, 25th, 75th, and 95th percentiles; AUC_ss_, area under the concentration–time curve at steady state; BW, baseline body weight for patient; CL, clearance; C_max_, peak concentration; C_trough_, trough concentration; P, percentile; Q, intercompartmental clearance; ss, steady state; V_1_, central volume of distribution; V_2_, peripheral volume of distribution.

**SUPPLEMENTARY TABLE 3 |** Double sigmoid E_max_ model forecasted values of free C5.

| Hemolysis (%) | 90% CI of Hemolysis (%)^a^ | Free C5 (μg/mL) |
| --- | --- | --- |
| 1 | -3.28, 5.28 | 0.181 |
| 2 | -2.28, 6.28 | 0.241 |
| 3 | -1.28, 7.28 | 0.277 |
| 4 | -0.28, 8.28 | 0.303 |
| 4.28 | 0, 8.56 | 0.309 |
| 5 | 0.72, 9.28 | 0.324 |
| 20 | 15.72, 24.28 | 0.510 |

^a^Calculated using 90% CI = Hemolysis ± 1.645*SD where SD is 2.6.

C5, complement protein 5; CI, confidence interval; SD, standard deviation.

**SUPPLEMENTARY FIGURE 1 |** Prediction-corrected visual predictive check of final pharmacokinetic model (maintenance phase).


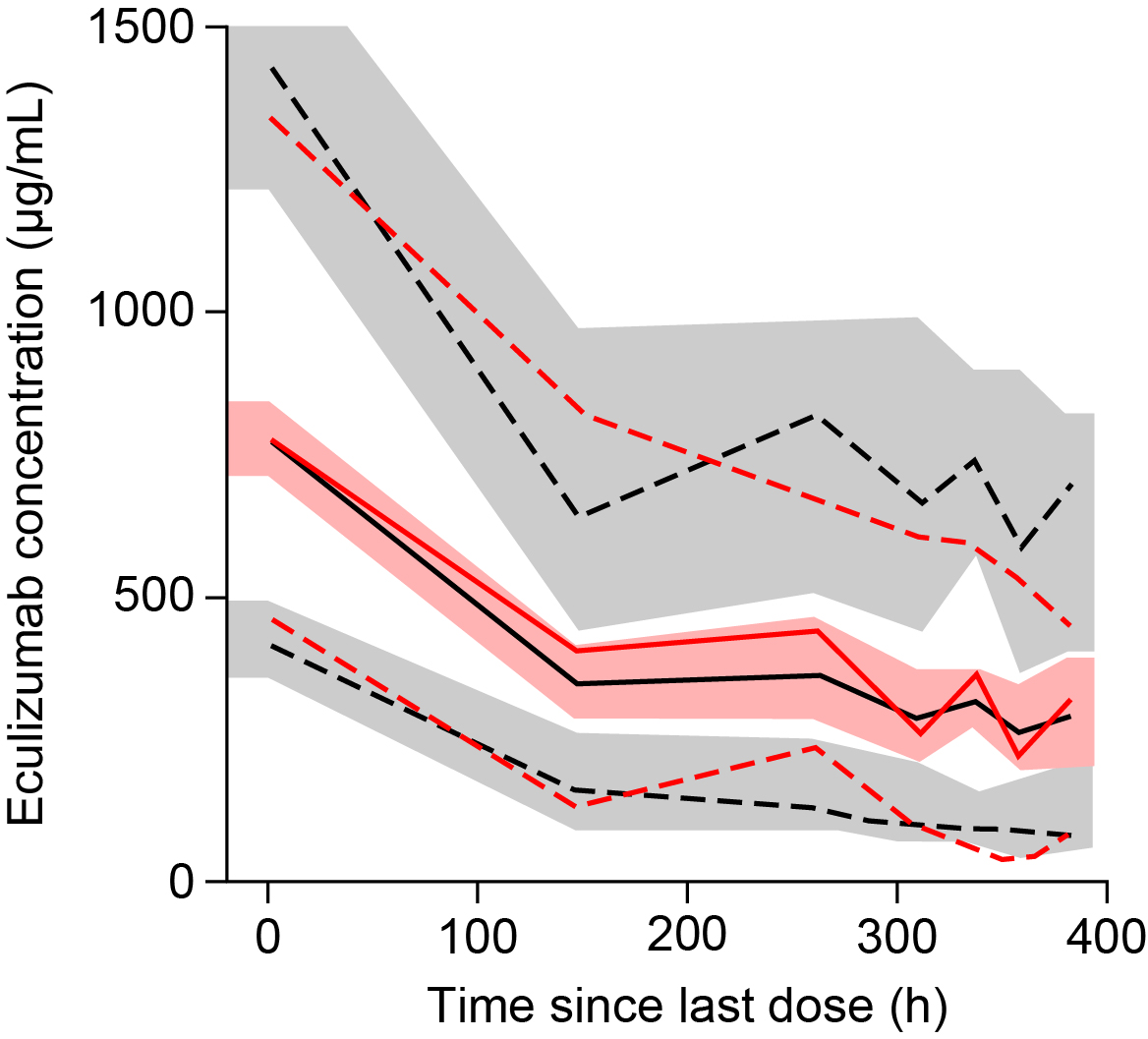


The red solid and dashed lines represent median and 5th–95th percentiles of the observed data. The black solid and dashed lines represent median and 5th–95th percentiles of the simulated data. Shaded areas represent the 90% confidence intervals of the simulated medians (red) and 5th–95th percentiles (gray).

**SUPPLEMENTARY FIGURE 2 |** Visual predictive check of final free C5 model.


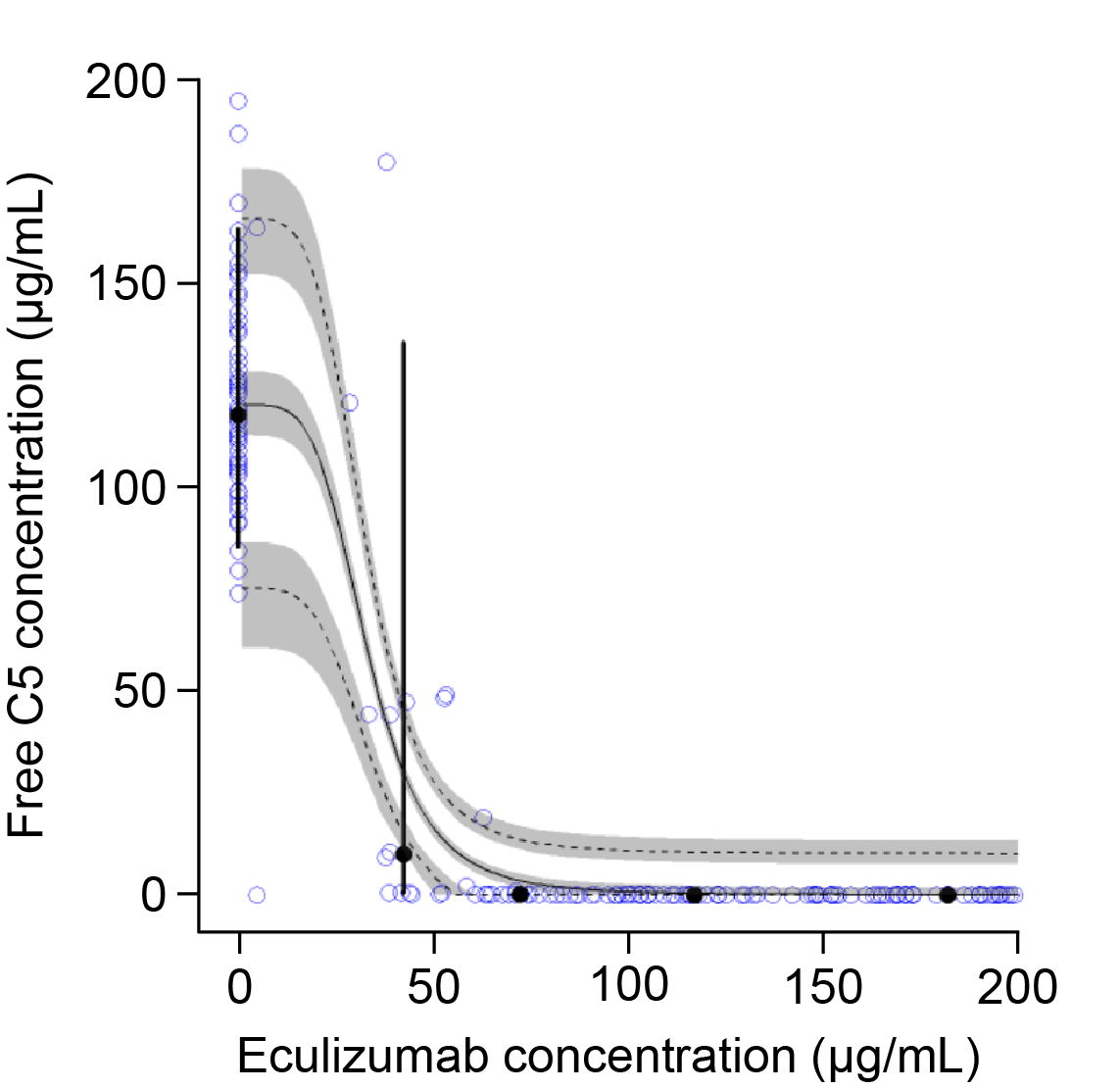


The solid line represents the model-predicted median free C5 concentration, the dotted lines the 5th and 95th percentiles of the model-predicted free C5 concentration, and the shaded gray areas the simulation-based 95% confidence interval for the median and 5th and 95th percentiles. The observations are represented by blue circles. The observed median and 5th and 95th percentiles of the free C5 concentration are represented by the error bars at the median eculizumab concentrations in the five concentration bins: 0 to <20 μg/mL (n=62), 20 to <60 μg/mL (n=16), 60 to <90 μg/mL (n=20), 90 to <150 μg/mL (n=42), and 150–200 μg/mL (n=44), with n the number of observations.

C5, complement protein 5.

**SUPPLEMENTARY FIGURE 3 |** Visual predictive check of the final hemolysis model.


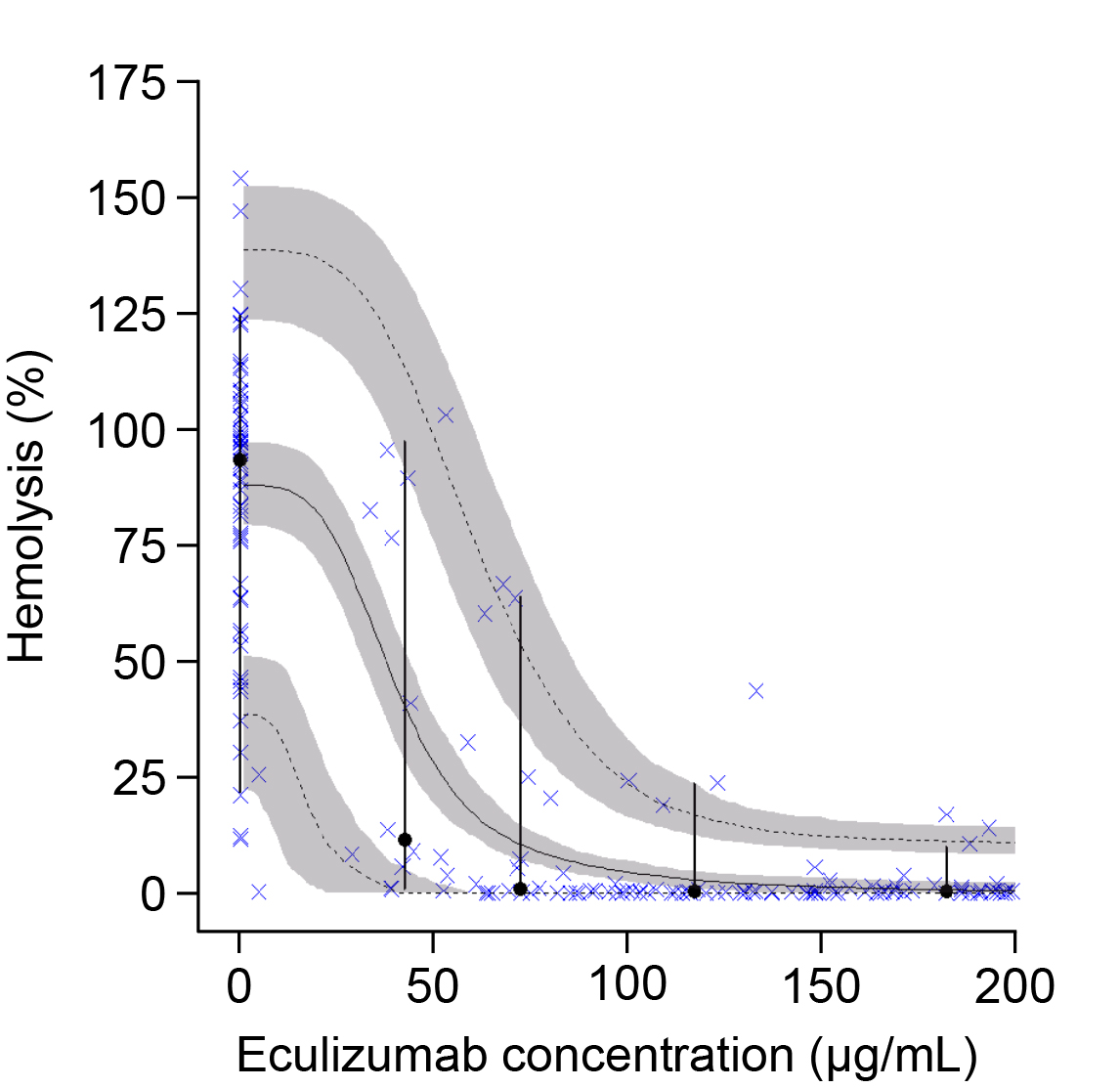


The solid line represents the model-predicted median percentage hemolysis, the dotted lines the 5th and 95th percentiles of the model-predicted percentage hemolysis, and the shaded gray areas the simulation-based 95% confidence interval for the median and 5th and the 95th percentiles. The observed median and 5th and 95th percentiles of the percentage hemolysis are represented by the error bars at the median eculizumab concentrations in the five concentration bins: 0 to <20 μg/mL (n=63), 20 to <60 μg/mL (n=16), 60 to <90 μg/mL (n=20), 90 to <150 μg/mL (n=42), and 150–200 μg/mL (n=43), with n the number of observations.

**SUPPLEMENTARY FIGURE 4 |** Modeled eculizumab exposure–response profile for serum free C5.


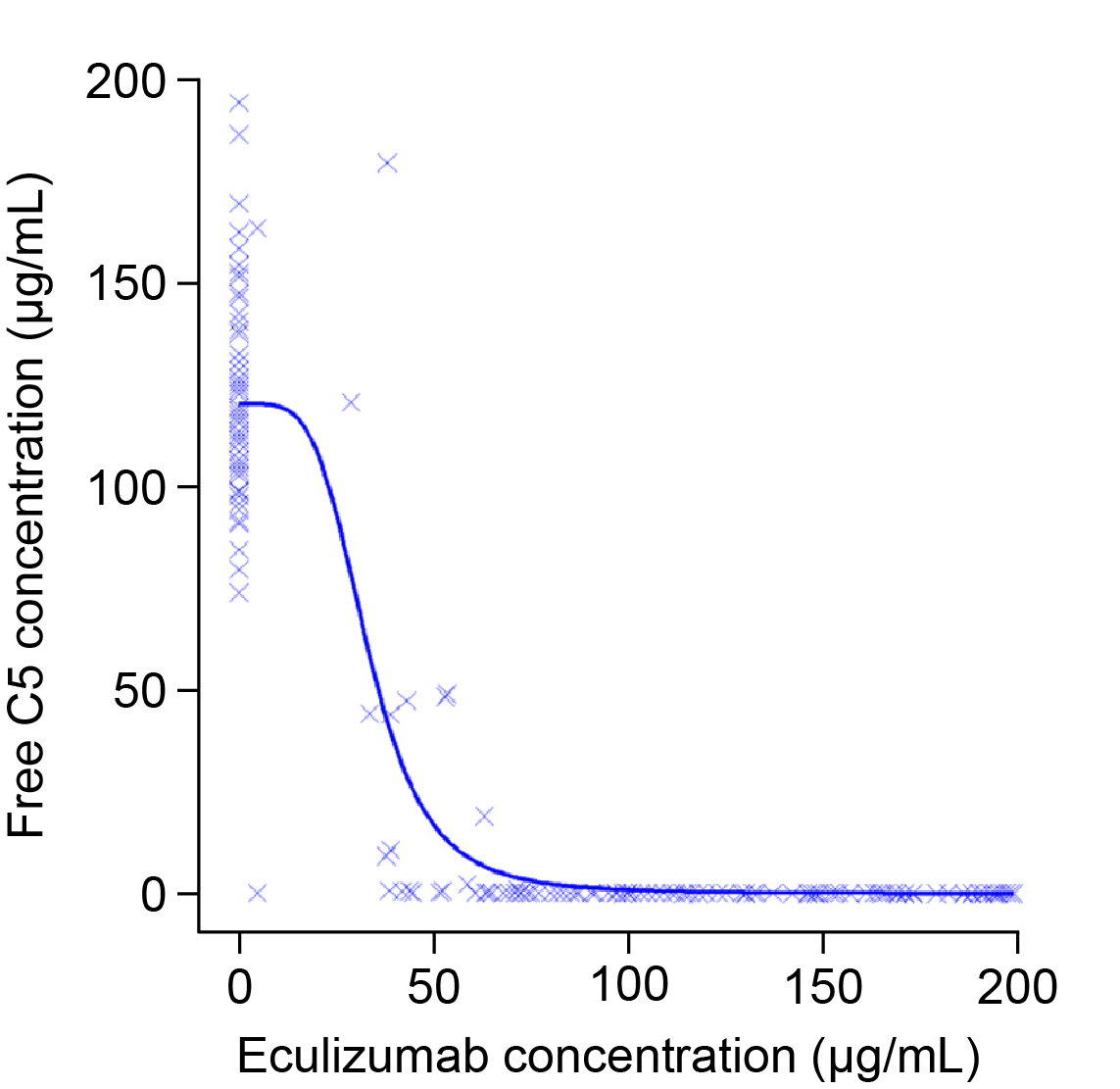


Free C5 concentration vs eculizumab concentration with the fitted regression curve from the final model.

C5, complement protein 5.

**SUPPLEMENTARY FIGURE 5 |** CWRES vs time after dose for the **(A)** one-compartment vs **(B)** two-compartment model.

CWRES, conditional weighted residuals.

**SUPPLEMENTARY FIGURE 6 |** In vitro cRBC hemolysis vs free C5 concentration fit with double sigmoid E_max_ model.


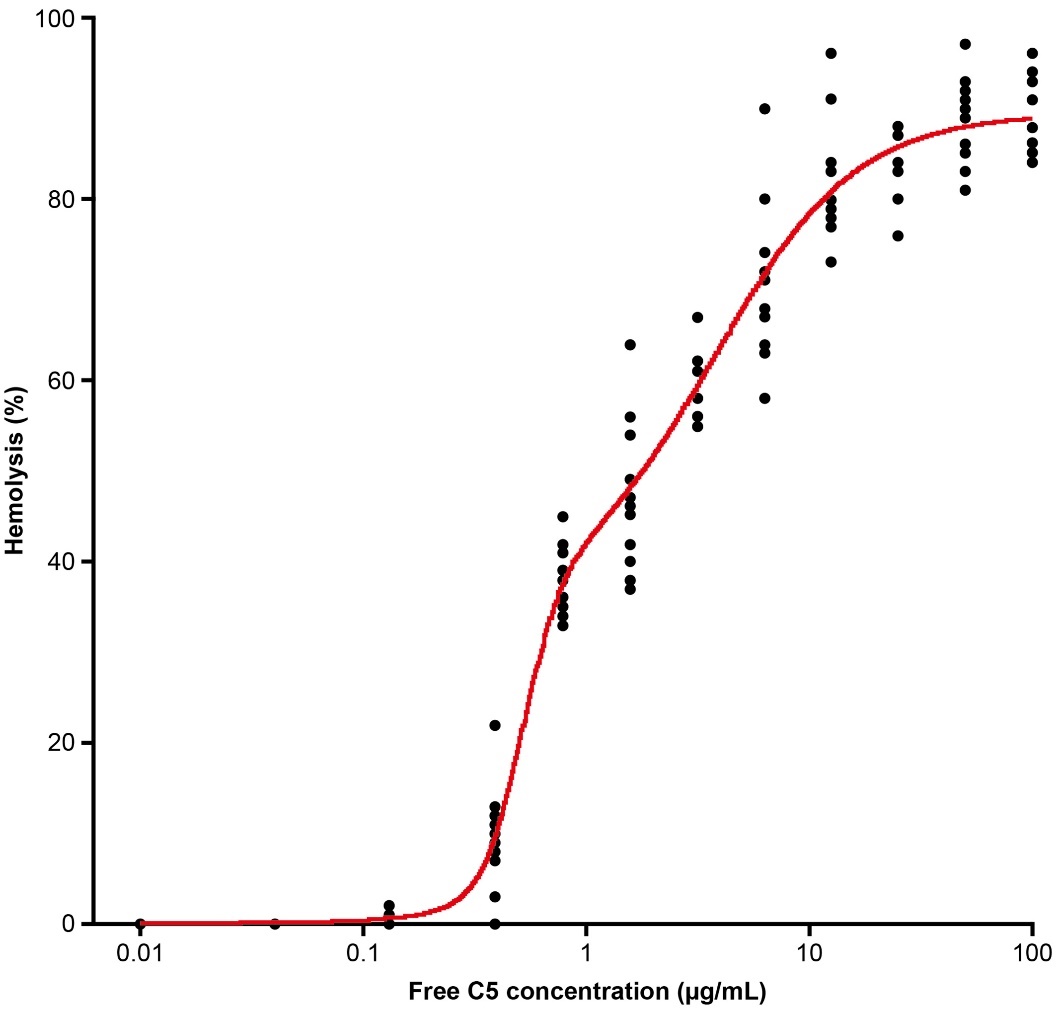


Symbols are observed in vitro results; the red line is the model-derived relationship.

C5, complement protein 5; cRBC, chicken red blood cell.
