# Supplementary material for: Eculizumab Pharmacokinetics and Pharmacodynamics in Patients With Generalized Myasthenia Gravis
Source: Front Neurol. 2021 Nov 2;12:696385. doi: 10.3389/fneur.2021.696385 (PMC8594444; doi:10.3389/fneur.2021.696385)
Supplement: Supplementary file 2 [file Data_Sheet_2.pdf]

**Supplementary Table: REGAIN study sites and ethics committee/institutional review boards for patients included in the PK/PD analysis**

| **Study site** | **Ethics committee/institutional review board (IRB)** |
|---|---|
| *ARGENTINA* | |
| Hospital Italiano, Buenos Aires, Argentina | Comité de Ética de Protocolos de Investigación (CEPI), Juan. D Perón 4190, C.A.B.A, Buenos Aires C1181 ACH, Argentina |
| Instituto de Investigaciones Neurológicas Raúl Carrea (FLENI), Buenos Aires, Argentina | Comite de Etica en Investigaciones Biomedicas, Montañeses 2325 Ciudad Autonoma, Buenos Aires C1428 AQK, Argentina |
| *BELGIUM* | |
| UZ Antwerpen, Edegem, Antwerp, Belgium | Ethisch Comité Universitair Ziekenhuis, Antwerpen, Wilrijkstraat 10, 2650 Edegem, Belgium |
| | Commissie Medische Ethiek, Algemeen Ziekenhuis Sint-Lucas, Groenebriel 1, 9000 Gent, Belgium |
| | Commissie Medische Ethiek van de Universitaire Ziekenhuizen K.U. Leuven, Campus Gasthuisberg E330, Herestraat 49, 3000 Leuven, Belgium |
| UZ Leuven, Leuven, Belgium | Ethisch Comité Universitair Ziekenhuis, Antwerpen, Wilrijkstraat 10, 2650 Edegem, Belgium |
| | Commissie Medische Ethiek, Algemeen Ziekenhuis Sint-Lucas, Groenebriel 1, 9000 Gent, Belgium |
| | Commissie Medische Ethiek van de Universitaire Ziekenhuizen K.U. Leuven, Campus Gasthuisberg E330, Herestraat 49, 3000 Leuven Belgium |
| AZ Sint-Lucas-Campus Sint-Lucas, Gent, Belgium | Ethisch Comité Universitair Ziekenhuis, Antwerpen, Wilrijkstraat 10, 2650 Edegem Belgium |
| | Commissie Medische Ethiek, Algemeen Ziekenhuis Sint-Lucas, Groenebriel 1, 9000 Gent Belgium |

| | |
|---|---|
| | Commissie Medische Ethiek van de Universitaire Ziekenhuizen K.U. Leuven, Campus Gasthuisberg E330, Herestraat 49, 3000 Leuven, Belgium |
| *BRAZIL* | |
| Fundação Faculdade Regional de Medicina de São José do Rio Preto, Sao Paulo, Brazil | CEP da Faculdade de Medicina de São José do Rio Preto – FAMERP, Av. Brigadeiro Faria Lima 5416, São José do Rio Preto, Sao Paulo 15090-000 Brazil |
| | Comissao Nacional de Etica em Pesquisa, Sepn 510 Norte, Bloco A 1º Subsolo, Edifício Ex-Inan - Unidade II - Ministério da Saúde Asa Norte, Brasilia, DF 70750-521, Brazil |
| Hospital Mãe de Deus, Rio Grande do Sul, Brazil | Comitê de Ética em Pesquisa em Seres Humanos, Associação Educadora São Carlos – Hospital Mae de Deus, Rua José de Alencar, 286 Porto Alegre, RS, 90880-480, Brazil |
| | Comissao Nacional de Etica em Pesquisa, Sepn 510 Norte, Bloco A 1º Subsolo, Edifício Ex-Inan - Unidade II - Ministério da Saúde Asa Norte, Brasilia, DF 70750-521, Brazil |
| Faculdade de Medicina do ABC, Sao Paulo, Brazil | CEP da Faculdade de Medicina do ABC, Avenida Príncipe de Gales 821. 1°andar, prédio CEPES, Santo André, Sao Paulo 09060-650, Brazil |
| | Comissao Nacional de Etica em Pesquisa, Sepn 510 Norte, Bloco A 1º Subsolo, Edifício Ex-Inan - Unidade II - Ministério da Saúde, Brasilia, DF 70750-521, Brazil |
| Universidade Federal de Sao Paulo, Sao Paulo, Brazil | CEP da Universidade Federal de São Paulo / Hospital São Paulo, Rua Botucatu, 572 - 1º andar, São Paulo, 04023-062, Brazil |
| | Comissao Nacional de Etica em Pesquisa, Sepn 510 Norte, Bloco A 1º Subsolo Edifício Ex-Inan - Unidade II - Ministério da Saúde, Brasilia, DF 70750-521, Brazil |
| *CANADA* | |
| University of Alberta Hospital, Edmonton, Alberta | University of Alberta Research Ethics Office, 308 Campus Tower, 8625 – 112 St. NW, Edmonton, Alberta T6G 1K8, Canada |

| | |
|---|---|
| *CZECH REPUBLIC* | |
| Vseobecna Fakultni Nemocnice v Praze, Praha, Czech Republic | Eticka komise Vseobecne fakultni nemocnice v Praze, Na Bojisti 1, III. Patro 128 08, Praha 2, Czech Republic |
| | Eticka komise Fakultni nemocnice Brno, Jihlavska 20, 625 00, Brno, Czech Republic |
| Fakultni Nemocnice Ostrava, Ostrava, Czech Republic | Eticka komise Fakultni Nemocnice Ostrava, 17. Listopadu 1790/5, 708 52 Ostrava-Poruba, Czech Republic |
| | Eticka komise Fakultni nemocnice Brno, Jihlavska 20, 625 00, Brno, Czech Republic |
| Fakultni Nemocnice Brno, Brno, Czech Republic | Eticka komise Fakultni nemocnice Brno, Jihlavska 20, 625 00, Brno, Czech Republic |
| *DENMARK* | |
| Aarhus Universitetshospital, Aarhus C, Denmark | De Videnskabsetiske Komitéer for Region Hovedstaden, Kongens Vænge 2, Hillerød, 3400 Denmark |
| Rigshospitalet, Copenhagen, Denmark | De Videnskabsetiske Komitéer for Region Hovedstaden, Kongens Vænge 2, Hillerød, 3400 Denmark |
| *FINLAND* | |
| Neuro NEO Oy, Turku, Finland | National Committee on Medical Research Ethics, Tukija, Valvira, P.O. Box 210, Helsinki, FI-00531, Finland |
| *HUNGARY* | |
| Jahn Ferenc Del-Pesti Korhaz es Rendelointezet, Budapest, Hungary | Medical Research Council Ethics Committee for Clinical Pharmacology, Arany János u.6-8., Budapest, H-1051 Hungary |
| Szegedi Tudomanyegyetem Szent-Gyorgyi Albert Klinikai Kozpont, Szeged, Hungary | Medical Research Council Ethics Committee for Clinical Pharmacology, Arany János u.6-8., Budapest, H-1051 Hungary |
| *ITALY* | |
| Azienda Ospedaliera Universitaria "Federico II", Naples, Italy | Comitato Etico per le attività biomediche "Carlo Romano" Università degli Studi di Napoli Federico II Via Pansini 5, Napoli, 80131 Italy |

| | |
|---|---|
| Policlinico Universitario Agostino Gemelli, Rome, Italy | Comitato Etico Univ. Cattolica del Sacro Cuore Policlinico A. Gemelli, Largo Agostino Gemelli 8, Roma, 00168, Italy |
| Azienda Ospedaliera Sant' Andrea – Universita di Roma La Sapienza, Rome, Italy | Comitato Etico Azienda Policlinico Umberto I, Viale del Policlinico 155, Roma, 00161 Italy |
| Fondazione IRCCS Instituto Neurologico "Carlo Besta", Milan, Italy | Comitato Etico Fondazione IRCCS Istituto Neurologico "Carlo Besta", Via Celoria 11, Milano, 20133 Italy |
| Umberto I Pol. Di Roma-Universita di Roma La Sapienza, Rome, Italy | Comitato Etico Azienda Policlinico Umberto I, Viale del Policlinico 155, Roma, 00161 Italy |
| *JAPAN* | |
| Kyushu University Hospital, Fukuoka, Japan | Kyushu University Hospital IRB, Higashi-ku Maidashi 3-1-1, Fukuoka-shi, Fukuoka-Ken 812-8582 Japan |
| Nagasaki University Hospital, Nagasaki, Japan | Nagasaki University Hospital IRB, Sakamoto 1-7-1, Nagasaki-shi, Nagasaki-Ken 852-8501 Japan |
| Chiba University Hospital, Chiba, Japan | Chiba University Hospital IRB, 1-8-1 Inohana, Chuo-ku, Chiba-shi, Chiba-Ken, 260-8677 Japan |
| Hanamaki General Hospital, Hanamaki, Japan | Hanamaki General Hospital IRB, Kajo-machi 4-28, Hanamaki-shi, Iwate-Ken 025-0075 Japan |
| NHO Sendai Medical Center, Sendai, Japan | NHO Sendai Medical Center IRB, Miyagino-ku Miyagino 2-8-8 Sendai-shi, Miyagi-Ken 983-8520 Japan |
| Sapporo Medical University Hospital, Sapporo, Japan | Sapporo Medical University Hospital IRB, 291 Minami 1jo Nishi 16-chome, Chuo-ku, Sapporo-shi, Hokkaido 060-8543 Japan |
| Kindai University Hospital, Osakasayama, Japan | Kindai University Hospital IRB, Onohigashi 377-2, Osakasayama-shi, Osaka-Fu 589-8511 Japan |
| Osaka University Hospital, Suita, Japan | Osaka University Hospital IRB, Yamadaoka 2-15, Suita-shi, Osaka-Fu 565-0871 Japan |

| | |
|---|---|
| *REPUBLIC OF KOREA* | |
| Samsung Medical Center, Seoul, Republic of Korea | IRB of Samsung Medical Center, 81 Irwon-ro, Gangnam-gu, Seoul, 135-710 Korea |
| Korea University Anam Hospital, Seoul, Republic of Korea | IRB of Korea University Anam Hospital, 73 Inchon-ro, Seongbuk-gu, Seoul, 136-705, Republic of Korea |
| Seoul Metropolitan Government Seoul National University, Seoul, Republic of Korea | IRB of Seoul Metropolitan Government Seoul National University Boramae Medical Center, 20 Boramae-ro 5-gil, Dongjak-gu, Seoul, 156-707 Korea |
| Severance Hospital, Yonsei University, Seoul, Republic of Korea | IRB of Severance Hospital, Yonsei University Health System, 50-1 Yonsei-ro, Seodaemun-gu, Seoul, 120-752 Korea |
| *NETHERLANDS* | |
| Academisch Medisch Centrum, Amsterdam, Netherlands | Medisch Ethische Toetsingscommissie (MEC) Academisch Medisch Centrum Amsterdam, Kamernummer: E2-170/172, Meibergdreef 9 1105 AZ Amsterdam, The Netherlands |
| *SPAIN* | |
| Hospital Universitari de Bellvitge, Barcelona, Spain | CEIC, Hospital Universitari Vall d'Hebron, Edificio Maternoinfantil, Planta 13, Paseo Vall d'Hebron 119-129 Barcelona 08035, Spain |
| | CEIC, Hospital Universitàrio de Bellvitge, Edifici Unitat de Recerca Feixa Llarga, s/n L'Hospitalet de Llobregat, Barcelona, 08907, Spain |
| Hospital Universitario La Paz, Madrid, Spain | Clinical Research Ethics Committee/Comite Etico de Investigacion Clinica, CEIC Hospital Universitàrio La Paz, Paseo de la Castellana 261, Planta 11, Neurology, Hospital General – Planta Madrid, Madrid 28046, Spain |
| | CEIC Hospital Universitàrio de Bellvitge, Edifici Unitat de Recerca Feixa Llarga, s/n L'Hospitalet de Llobregat, Barcelona, 08907, Spain |
| Hospital Universitari Vall d'Hebron, Barcelona, Spain | CEIC Hospital Universitari Vall d'Hebron, Edificio Maternoinfantil, Planta 13, Paseo Vall d'Hebron 119-129 Barcelona 08035, Spain |
| | CEIC Hospital Universitàrio de Bellvitge, Edifici Unitat de Recerca Feixa Llarga, s/n L'Hospitalet de Llobregat, Barcelona, 08907, Spain |

| | |
|---|---|
| Hospital de la Santa Creu i Sant Pau, Barcelona, Spain | Hospital de la Santa Creu I Sant Pau Servicio de Farmacología Clínica – Pabellón, 18 Av. Sant Antoni Mª Claret 167, Barcelona, 08025, Spain |
| | Comite Etico de Investigacion Clinica (CEIC), Hospital Universitàrio de Bellvitge, Edifici Unitat de Recerca Feixa Llarga, s/n L'Hospitalet de Llobregat, Barcelona, 08907, Spain |
| *SWEDEN* | |
| Karolinska Universitetssjukhuset, Stockholm, Sweden | Regionala Etikprövningsnämnden i Stockholm Karolinska Institutet/Solna, Nobels vägn 9, plan 3D, SE-171 65 Stockholm, Sweden |
| *TURKEY* | |
| Kocaeli Universitesi, Kocaeli, Turkey | Ege Üniversitesi Tip Fakültesi Klinik, Araştirmalar Etik Kurulu, Ege Üniversitesi Tip Fakültesi Dekanliği 2, Kat Erzene Ankara Caddesi, Bornova 35100, Izmir, Turkey |
| Hacettepe Universitesi Tip Fakultesi, Ankara, Turkey | Ege Üniversitesi Tip Fakültesi Klinik, Araştirmalar Etik Kurulu, Ege Üniversitesi Tip Fakültesi Dekanliği 2, Kat Erzene Ankara Caddesi, Bornova 35100, Izmir, Turkey |
| Dokuz Eylul Univrsitesi Tip Fakultei, Izmir, Turkey | Ege Üniversitesi Tip Fakültesi Klinik Araştirmalar Etik Kurulu, Ege Üniversitesi Tip Fakültesi Dekanliği 2, Kat Erzene Ankara Caddesi, Bornova 35100, Izmir, Turkey |
| Ondokuz Mayis University of Medicine, Samsun, Turkey | Ege Üniversitesi Tip Fakültesi Klinik, Araştirmalar Etik Kurulu, Ege Üniversitesi Tip Fakültesi Dekanliği 2, Kat Erzene Ankara Caddesi, Bornova 35100, Izmir, Turkey |
| *UNITED KINGDOM* | |
| The Walton Centre NHS Foundation Trust, Liverpool, Merseyside, UK | The Walton Centre NHS Foundation Trust Clinical Trials Unit, 1st Floor, Lower Lane, Fazakerley, Liverpool, Merseyside L9 7LJ, United Kingdom |
| Queen Elizabeth Hospital, Birmingham, West Midlands, UK | NRES Committee East Midlands – Leicester, The Old Chapel, Royal Standard Place, Nottingham, NG1 6FS, United Kingdom |

| | |
|---|---|
| King's College Hospital, London, UK | NRES Committee East Midlands – Leicester, The Old Chapel - Royal Standard Place, Nottingham, NG1 6FS, United Kingdom |
| *UNITED STATES OF AMERICA* | |
| Ohio State University Medical Center, Columbus, Ohio, USA | Western Institutional Review Board (WIRB), 1019 39th Avenue SE, Suite 120, Puyallup, WA 98374-2115, USA |
| University of Kansas Medical Center Research Institute Inc, Kansas City, Kansas, USA | Human Subjects Committee University of Kansas Medical Center, 3901 Rainbow Boulevard, Kansas City, Kansas 66160, USA |
| University of Maryland Medical Center, Baltimore, Maryland, USA | University of Maryland, Baltimore Institutional Review Board, 800 W. Baltimore Street, Suite 100, Baltimore, Maryland 21201, USA |
| Johns Hopkins University School of Medicine, Baltimore, Maryland, USA | JHM Office of Human Subjects Research – Institutional Review Boards, 1620 McElderry Street, Baltimore, Maryland 21205-1911, USA |
| Wesley Neurology Clinic, Cordova, Tennessee, USA | Copernicus Group IRB, 1 Triangle Drive, Suite 100, Durham, North Carolina 27713, USA |
| University of Florida at Shands, Jacksonville, Florida, USA | Western Institutional Review Board (WIRB), 1019 39th Avenue SE, Suite 120, Puyallup, WA 98374-2115, USA |
| University of North Carolina at Chapel Hill, Chapel Hill, North Carolina, USA | Office of Human Research Ethics, University of North Carolina at Chapel Hill Medical School, Building 52, CB# 7097, Chapel Hill, North Carolina 27599, USA |
| University of California San Francisco-Fresno, Fresno, San Francisco, USA | Community Medical Centers Institutional Review Board, 155 N. Fresno Street, Suite 290, Fresno, CA 93701, USA |
| Duke University Health System, Durham, North Carolina, USA | Duke University Health System Institutional Review Board, 2424 Erwin Road, Suite 405, Campus Box 2712 Durham, North Carolina 27705, USA |
| University of Vermont Medical Center, Burlington, Vermont, USA | University of Vermont Committee on Human Research in the Medical Sciences, 213 Waterman Building, 85 South Prospect Street, Burlington, Vermont 05405, USA |
| Indiana University, Indianapolis, Indiana, USA | Indiana University Institutional Review Board, 980 Lockfield Village, Third Floor, Indianapolis, Indiana 46202, USA |

| | |
|---|---|
| University of Iowa College of Medicine, Iowa City, Iowa, USA | Western Institutional Review Board (WIRB), 1019 39th Avenue SE, Suite 120, Puyallup, WA 98374-2115 USA |
| Brigham and Women's Hospital, Boston, Massachusetts, USA | Partners Human Research Committee, 116 Huntington Avenue, Suite 1002, Boston, Massachusetts 02116, USA |
| Oregon Health and Science University, Portland, Oregon, USA | Oregon Health and Science University IRB, 2525 SW 1st Ave, Suite 125 L106-RI, Portland, Oregon 97201, USA |
| University of California-Irvine, Irvine, California, USA | Institutional Review Board – Office of Research Administration,<br>5171 California Avenue, Suite 150, Irvine, California 92697 USA |
| Lahey Clinic Medical Center, Burlington, Massachusetts, USA | Lahey Hospital and Medical Center IRB, 41 Mall Road, Burlington, Massachusetts 01805, USA |
| University of Washington, Seattle, Washington, USA | Western Institutional Review Board (WIRB), 1019 39th Avenue SE, Suite 120, Puyallup, WA 98374-2115, USA |
| University of Southern California, Los Angeles, California, USA | USC Health Sciences Institutional Review Board, 1200 North State Street, Suite 4700, Los Angeles, California 90033, USA |
| The University of Texas Health Science, San Antonio, Texas, USA | The University of Texas Health Science Center, San Antonio IRB, 7703 Floyd Curl Drive, San Antonio, Texas 78229, USA |
| Southern Illinois University School of Medicine, Springfield, Illinois, USA | Springfield Committee for Research Involving Human Subjects, 801 N. Rutledge Street, Springfield, Illinois 62794, USA |
| University of Miami School of Medicine, Miami, Florida, USA | University of Miami Institutional Review Boards, Human Subject Research Office, 1500 NW 12th Avenue, Suite 1002, Miami, Florida 33136 ,USA |
| UT Southwestern Medical Center, Dallas, Texas, USA | University of Texas Southwestern Medical Center Investigational Review Board, 5323 Harry Hines Blvd., Dallas, Texas 75390, USA |
| University of South Florida, Tampa, Florida, USA | Western Institutional Review Board (WIRB), 1019 39th Avenue SE, Suite 120, Puyallup, WA 98374-2115, USA |
| Yale University, New Haven, Connecticut, USA | Human Research Protection Program (HRPP), 55 College Street, New Haven, Connecticut 06510, USA |
| University of Alabama, Birmingham, Alabama, USA | Western Institutional Review Board (WIRB), 1019 39th Avenue SE, Suite 120, Puyallup, WA 98374-2115, USA |

| | |
|---|---|
| Las Vegas Clinic, Las Vegas, Nevada, USA | Copernicus Group IRB, 1 Triangle Drive, Suite 100, Durham, North Carolina 27713, USA |
| California Pacific Medical Center, San Francisco, California, USA | Western Institutional Review Board (WIRB), 1019 39th Avenue SE, Suite 120, Puyallup, WA 98374-2115, USA |
| Stanford University School of Medicine, Stanford, California, USA | Stanford IRB, Research Compliance Office, 3000 El Camino Real; Five Palo Square, 4th Floor, Palo Alto, California 94306, USA |
| Carolinas Healthcare System, Charlotte, North Carolina, USA | Copernicus Group IRB, 1 Triangle Drive, Suite 100, Durham, North Carolina 27713, USA |
